# Predictive metabolite signatures for risk of progression to active TB from QuantiFERON supernatants of household contacts of TB patients

**DOI:** 10.1080/22221751.2024.2437242

**Published:** 2024-12-04

**Authors:** Evangeline Ann Daniel, Shubham Upadhyay, Murugesan Selvachithiram, Sathyamurthi Pattabiraman, Brindha Bhanu, Amsaveni Sivaprakasam, Vandana Kulkarni, Rajesh Karyakarte, Sanjay Gaikwad, Mandar Paradkar, Shri Vijay Bala Yogendra Shivakumar, Vidya Mave, Amita Gupta, Keshava Prasad, Luke Elizabeth Hanna

**Affiliations:** a Department of Virology and Biotechnology, National Institute for Research in Tuberculosis, Indian Council of Medical Research (ICMR), Chennai, India; b University of Madras, Chennai, India; c Center for Systems Biology and Molecular Medicine, Yenepoya University, Mangalore, India; d BJ Government Medical College-Johns Hopkins Clinical Research Site, Pune, India; e Johns Hopkins Center for Infectious Diseases in India, Pune, India; f Byramjee Jeejeebhoy Government Medical College and Sassoon General Hospitals, Pune, India; g Johns Hopkins University School of Medicine, Baltimore, MD, USA

**Keywords:** Metabolites, tuberculosis, progression, QuantiFERON supernatant, biomarkers, metabolite signatures, diagnosis

## Abstract

The identification of individuals with the greatest risk of progression to active tuberculosis (TB) disease from the huge reservoir of *Mycobacterium tuberculosis* (*Mtb*) infected individuals continues to remain an arduous ascent in the global effort to control TB. In a two-year prospective study, we analysed metabolic profiles in the unstimulated and TB antigen stimulated QuantiFERON supernatants of 14 healthy household contacts (HHCs) who progressed to TB disease (Progressors) and 14 HHCs who remained healthy (Non-Progressors). We identified 21 significantly dysregulated metabolites in the TB antigen-stimulated QuantiFERON supernatants of Progressors, of which the combination of Malic acid and N-Arachidonoylglycine had maximum AUC of 0.99. Eighteen significantly dysregulated metabolites were identified in the unstimulated QuantiFERON supernatants of Progressors, among which the combination of Orotic acid and the phosphatidylcholines PC (O-34:1), PC (O-18:1(9Z)/16:0), PC (O-18:1(11Z)/16:0) had the maximum AUC of 0.98. Most of the dysregulated metabolites belonged to the pathways of fatty acid metabolism, lipid metabolism and nitric oxide metabolism. Validation of these metabolic signatures in large, diverse cohorts would pave way for the development of a robust test that can identify individuals at high risk of TB for targetted intervention of TB disease.

## Introduction

It is estimated that approximately 25% of the world’s population is infected with *Mycobacterium tuberculosis* and 5–10% of this fraction bear the risk of progressing to the active tuberculosis disease (TB) [1,2]. Systematic screening of contacts and high-risk groups and orienting them for treatment is an essential element of the End TB strategy put forth by the World Health Organization (WHO) [3]. The tests available at present for diagnosing TB infection *viz*. Interferon Gamma Release Assay (IGRA) and Tuberculin Skin Test (TST) have a low positive predictive value (PPV) [4], compromised sensitivity and specificity [5], and cannot classify the dynamic tuberculosis disease spectrum [6]. Implementing TST or IGRA-based screening and preventive treatment in countries where TB is endemic would necessitate treating 50–80% of the population, the majority of whom would not actually require treatment [7]. Hence, research has shifted towards discovering non-sputum-based biomarkers and biosignatures with improved diagnostic performance that can easily be translated as point-of care tests.

With advancements in technology, several omics-based platforms have become available for biomarker identification. Metabolomics is the characterization of small molecules (<1000 Da) that are the downstream products of diverse cellular processes in the biological system. Disease states can disrupt the metabolic pathway of the host and cause dysregulation of metabolites, which can be quantitatively measured in biofluids [8]. *Mycobacterium tuberculosis* infection can cause significant alterations in host protein, lipid and energy metabolism, which can be detected in blood, making small metabolites potential biomarkers. Studies investigating the metabolic alterations and resulting altered metabolite profile can throw light on the complex host–pathogen interactions in TB infection and disease and help understand some of the key features in every step of the continuum of the TB spectrum. A recent review has beautifully summarized the various biomarkers that have been identified for tuberculosis using the metabolomics approach [9]. However, there are very few studies in the list that have identified metabolic biomarkers for predicting the onset of disease [10–12]. We therefore aimed to analyse the metabolomic profile of healthy household contacts of TB disease patients who progressed to TB during a two-year follow-up study (Progressors) and compare it with that of individuals who did not progress to disease during follow-up (Non-progressors), to identify a metabolic signature that predicts the risk of progression to infectious TB disease. We, for the first time used QuantiFERON supernatants for the metabolic profiling to ensure identification of a TB-specific metabolite signature.

## Materials and methods

### Ethical approval

The study was conducted with the approval of the Institutional Ethics Committees of ICMR-National Institute for Research in Tuberculosis (ICMR-NIRT), Chennai, India, Byramjee Jeejeebhoy Government Medical College (BJGMC), Pune, India and Johns Hopkins University (JHU), Baltimore, Maryland USA. (IEC No. 2020021).

### Study cohort

A cohort of healthy household contacts (HHCs) of individuals newly diagnosed with pulmonary tuberculosis (PTB) was established and followed up from August 2014 to December 2017 as part of the Cohort for Tuberculosis Research by the Indo-US Medical Partnership (C-TRIUMPH) study at two locations, ICMR-NIRT and BJGMC in India, in collaboration with JHU, USA. The study design and implementation details for C-TRIUMPH have been previously described [13]. Household contacts were defined as adults and children who lived in the same house as a TB patient during the three months preceding the patient's TB diagnosis. All household contacts underwent clinical and laboratory assessments at baseline, as well as at 4–6, 12 and 24 months to rule out TB. TST and IGRA were conducted at baseline and repeated at subsequent visits if the previous results were negative. The term “Progressors” was used to identify HHCs who developed TB at any time after 2 months of TB diagnosis in the index case. Confirmation of active TB disease in a household contact required a positive result in TB culture or GeneXpert/MTB Rif. Non-Progressors were defined as HHCs who stayed healthy and did not develop TB throughout the two-year follow-up period.

### Sample preparation for LC MS/MS

Metabolite extraction was performed using the triple solvent method [14] with certain modifications. In brief, LCMS-grade solvents acetonitrile, methanol, and water were used in a 2:2:1 ratio to prepare the triple solvent mixture. 50 µL of QuantiFERON supernatant was added to 900 µL of the triple solvent mixture and vortexed for 15 min. This was followed by sonication for 15 min. The sonicated mixture was incubated at −20°C overnight for protein precipitation. On the following day, the mixture was centrifuged at 12,000 rpm at 4°C for 20 min. The supernatant was transferred to a new 1.5 ml vial and vacuum-dried using a SpeedVac concentrator. The samples were resuspended in 250 µL of 0.1% formic acid and further diluted 3 times before acquisition. Epicatechin was used as the internal standard and was spiked in the samples at a concentration of 100 ng/mL.

### Data acquisition

Metabolites extracted from the supernatants were analysed using liquid chromatography followed by MS/MS on the QTRAP 6500 mass spectrometer (ABSciex) coupled with Agilent 1290 infinity II liquid chromatography system with a C18 RRHD Zorbax column (20×150 mm, 1.8 μm particle size). Data acquistion was carried out using Analyst Software, version 1.6.3 and the parameters for the analysis were set up using the Analyst Device Driver. A 25-minute Liquid Chromatography (LC) method was used to separate the metabolites. Two solvents were used: 0.1% formic acid in LCMS grade water (Solvent A) and 0.1% formic acid in 90% LCMS grade acetonitrile (Solvent B). The injection volume was set to 10 µL while the flow rate was set to 0.25 mL/min. The LC run was set for 25 min with the following gradient: 2% B for 1–10 min, 30% B for 10–14 min, 60% B for 14–18 min, 95% B for 18–21 min and 2% B for 21–25 min. Mass spectrometry data was collected using the information-dependent acquisition (IDA) method in low mass mode. The IDA method employed enhanced mass spectra (EMS) followed by enhanced product ion (EPI) mode. The top five spectra from the EMS mode were analysed in the EPI (MS/MS) mode using high-energy collision-induced dissociation (CID). Metabolite data was obtained in both positive (4500 V) and negative (−4500 V) polarities with a probe temperature of 450°C. The compound parameters, *viz*. declustering potential (DP) and collision energy (CE) were set to 100 and 10 V respectively and the injection volume was set to 10 µL. The samples were pooled in equal volumes to prepare the Quality Control (QC) sample. The QC samples were acquired at the start and at the end of the acquisition.

### Metabolomics data analysis

The raw files were converted to .mzml format using MSConvert [15]. The converted files were imported in MZmine 2.53 [16]. Feature detection was carried out with an m/z tolerance of 0.5 Da. This was followed by chromatogram deconvolution using the Noise Amplitude Algorithm with amplitude of noise as 1.5E2. Isotopes were detected using an Isotopic Peak Grouper with an m/z tolerance of 0.25 Da and retention time tolerance of 0.2 min. Join Aligner with an m/z tolerance of 0.05 Da and a retention time tolerance of 0.5 min was used to align the peaks. After processing, the data was searched against the Human Metabolome Database (HMDB) using the MS2 compound software [17,18]. Metabolite identification and annotation were carried out with a precursor mass tolerance of 0.05 Da. Adducts that were used for the positive mode were [M+H], [2M+H] and [M+2H] while, for the negative mode, [M-H], [2M-H] and [M-2H] were used.

### Statistical analysis

Feature filtering was performed using the Interquartile Range (IQR) to filter out the metabolites with near constant intensities across the sample conditions. The peak areas of metabolites were normalized using quantile normalization, log transformed and auto scaled before performing any statistical analysis. The normalized data was taken up for fold change analysis and Principal Component Analysis (PCA). The differentially expressed metabolites with a fold change of >1.3 and <0.76 and with *p*-values <0.05 as identified by the parametric, unpaired two sample t-test were considered as significantly dysregulated. Over Representation Analysis (ORA) for pathways and biomarker analysis were carried out using Metaboanalyst 6.0[19].

### Multivariate biomarker analysis

The “Biomarker Analysis” module from Metaboanalyst software [19] was used for multivariate biomarker analysis. Classification and feature ranking were performed using the Random forest method.

## Results

### Characteristics of the study cohort

**Figure 1.**
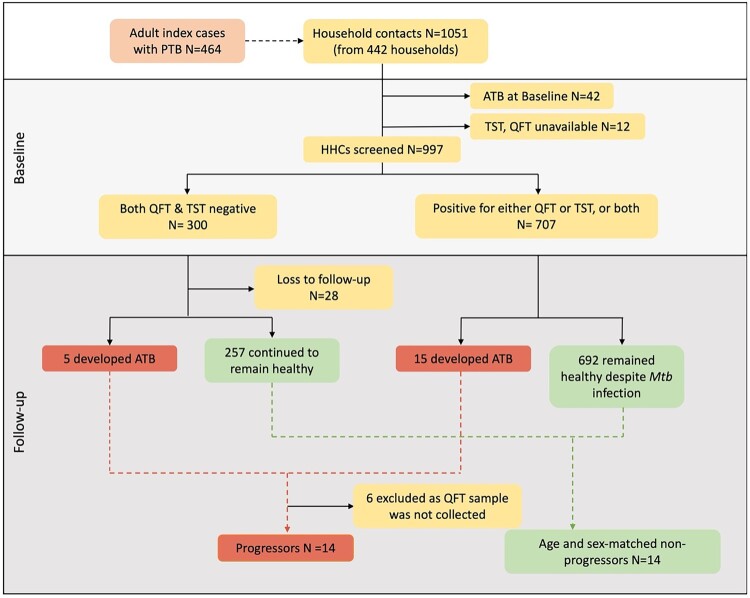
Participant selection from two sites of the C-TRIUMPh cohort study. A total of 1051 adults and children were recruited in the C-TRIUMPh study. Participants were classified based on their baseline Mtb-infection status as positive for QFT (≥0.35 IU/ml) and/or positive for TST (induration diameter ≥5 mm) or negative for both. Among the enrolled participants, those who went on to develop TB during follow-up were identified as Progressors, and those who remained healthy were defined as Non-progressors. Progressors were matched to Non-progressors for age and gender.

The clinical characteristics of the participants who contributed samples for the metabolite analysis are provided in Table 1. Among the 1051 HHCs from 442 households who were enrolled and followed-up for two years as part of the parent study, twenty individuals developed microbiologically confirmed TB within 2 years and were identified as Progressors; 6 of them had to be omitted from the study since they did not have stored QuantiFERON supernatants. In the Progressors, the time between enrolment and diagnosis of active TB ranged from 3 to 21 months. 14 HHCs who remained healthy during follow-up were matched for age and gender with the Progressors and were selected as controls (Non-progressors) for the study. Metabolic profiling was performed in the stimulated and unstimulated QuantiFERON supernatants collected at baseline (enrolment) for Non-progressors, at the timepoint closest to TB activation for Progressors. Figure 1 provides the flow chart depicting the selection of study participants.

**Table 1.** Clinical and demographic characteristics of the study population

	Non-Progressors (*N *= 14)	Progressors (*N *= 14)	*p*-Value
**Age in years**	31.5 (24.0, 38.0)	32.0 (24.0, 38.0)	0.812
**Gender**			
Male	5 (35.7%)	5 (35.7%)	>0.995
Female	9 (64.3%)	9 (64.3%)	
**BMI**	24.4 (20.2, 28.5)	21.7 (17.5, 26.1)	0.193
**BMI classification**			
BMI: <18.50	2 (14.3%)	5 (35.7%)	0.507
BMI: 18.50–24.99	7 (50.0%)	5 (35.7%)	
BMI: ≥25.0	5 (35.7%)	4 (28.6%)	
**Currently smoking**			
No	13 (92.9%)	14 (100.0%)	>0.995
Yes	1 (7.1%)	0 (0.0%)	
**Ever smoked**			
No	14 (100.0%)	13 (92.9%)	>0.995
Yes	0 (0.0%)	1 (7.1%)	
**Alcohol consumer**			
No	10 (71.4%)	11 (78.6%)	>0.995
Yes	4 (28.6%)	3 (21.4%)	
**HIV**			
Negative	14 (100.0%)	13 (92.9%)	>0.995
Positive	0 (0.0%)	1 (7.1%)	
**Diabetes**	0 (0.0%)	0 (0.0%)	
**TST in mm**	5.0 (4.0, 8.0)	6.0 (4.0, 10.0)	0.839
**Tuberculin skin test (TST) classification**			
TST: <5mm	5 (35.7%)	4 (30.8%)	>0.995
TST: ≥5mm	9 (64.3%)	9 (69.2%)	
TST: <10mm	11 (78.6%)	9 (69.2%)	0.678
TST: ≥10mm	3 (21.4%)	4 (30.8%)	
Interferon gamma release assay (IGRA) result			
Negative	4 (28.6%)	6 (42.9%)	0.695
Positive	10 (71.4%)	8 (57.1%)	
**Bacillus Calmette-Guerin (BCG)**			
No	8 (57.1%)	4 (30.8%)	0.252
Yes	6 (42.9%)	9 (69.2%)	

Continuous variables are presented as Median (First Quartile, Third Quartile) and tested using Mann-Whitney Test.

Categorical variables are presented as frequency (percentages) and tested using Fishers Exact Test.

TST – Monteux test results, BMI – Body Mass Index, NA – Not applicable.

*One of the progressors lacked TST results and was excluded from TST-related analysis.

### Metabolite feature extraction

Untargeted metabolomics was performed to identify the differentially expressed metabolites in the stimulated and unstimulated supernatants of the Progressors and Non-progressors. The features across the Progressor and Non-progressor groups for both the stimulated and unstimulated conditions were aligned together and extracted in both polarities (negative and positive) using MzMine 2.53. This resulted in the identification of 1139 and 11444 features in positive and negative modes respectively in the stimulated supernatants, and 730 and 4504 features in the negative and positive modes in the unstimulated supernatants respectively. PCA plots were generated for both positive and negative mode acquisitions along with the pooled QC samples. The PCA plot revealed clustering of the QC samples together. The unstimulated QC samples and the stimulated QC samples clustered separately as expected. The clustering of the QC samples confirmed technical reproducibility and consistent performance of the acquisitions.

### Differences in metabolic profile seen in TB antigen-stimulated QuantiFERON supernatants of Progressors and Non-progressors

To investigate TB-induced metabolic dysregulation, we first compared the metabolite abundance in TB antigen-stimulated QuantiFERON supernatants of Progressors with that of the Non-progressors. A total of 412 metabolites were putatively identified in the positive mode, out of which 23 were found to be significantly altered between the two groups with *p *< 0.05. In the negative mode, 377 metabolites were putatively identified, among which 10 were significantly dysregulated between the groups. Since we were only interested in the endogenous metabolic alterations, we excluded drug moieties and synthetic compounds that were identified by the database, except nicotine derivatives to see if smoking contributed to accelerated risk of progression/reactivation to TB. After filtering, a total of 22 metabolites (18 in the positive mode and 4 in the negative mode) were identified as significantly dysregulated metabolites. DG (18:2n6/0:0/20:3n6), a diglyceride compound exhibited the highest fold change of 3.5443.

### Differences in metabolic profile seen in unstimulated QuantiFERON supernatants of Progressors and Non-progressors

We further compared the differential abundance of metabolites in the unstimulated QuantiFERON supernatants of Progressors and Non-progressors and identified a total of 593 putative metabolites (343 in the positive mode and 250 in the negative mode), out of which 38 were significantly dysregulated (*p* value <0.05, 27 in the positive mode and 11 in the negative mode). After filtering out drug molecules and synthetic compounds, we found a total of 18 significantly dysregulated metabolites, 13 in positive mode and 5 in negative mode. We observed that fatty acids including Palmitelaidic acid, Hypogeic acid and Palmitoleic acid were highly abundant in Progressors (with a fold change of 7.5715) as compared to Non-progressors. Iodine was also significantly elevated in Progressors, with a fold change of 6.869. We also observed that diglycerides DG (14:1(9Z)/16:1(9Z)/0:0) and DG (16:1(9Z)/14:1(9Z)/0:0), were present in increased abundance in Progressors as compared to Non-progressors (with a fold change of 4.202), as observed in the stimulated QuantiFERON supernatants. Table 2 provides the list of significantly dysregulated metabolites in the QuantiFERON supernatants of Progressors compared to Non-progressors.

**Table 2.** Significantly dysregulated metabolites in the stimulated and unstimulated QuantiFERON supernatants of Progressors vs. Non-progressors.

Name of metabolite	Fold change	*p*-value
**Stimulated-Positive mode**		
N-Acetylserotonin sulphate	3.10	0.0033
3, 5-Tetradecadiencarnitine	3.04	0.0345
N,N,N-Trimethyl-L-alanyl-L-proline betaine; Leucylproline; Isoleucylproline	2.97	0.0262
Dihydroresveratrol 4'-sulfate; Dihydroresveratrol 3-sulfate	2.50	0.0106
7-Methylguanosine	2.30	0.0001
Pyroglutamine; Dihydrothymine	2.11	0.0370
Dimethyldithiophosphate	1.98	0.0004
Diethanolamine	1.77	0.0147
Oxalic acid	1.74	0.0272
Nitrate	1.60	0.0215
3-Methoxy-4-hydroxyphenylglycol glucuronide	1.58	0.0213
6-Methylthiopurine 5'-monophosphate ribonucleotide	1.58	0.0136
Butanol	1.43	0.0334
PS(20:3(8Z,11Z,14Z)/18:0); PS(18:0/20:3(8Z,11Z,14Z))	0.66	0.0330
Pelargonic acid	0.64	0.0049
4,8-Methano-8aH-bisbenzofuro[3,2-e:2’,3'-g]isoquinoline-1,8a-diol,7-(cyclopropylmethyl)-5,6,7,8,9,14b-hexahydro-, (4bS,8R,8aS,14bR)-	0.64	0.0085
N-Methylnicotinium	0.38	0.0024
**Stimulated-Negative mode**		
Behenic acid	3.03	1.5991
DG(18:2n6/0:0/20:3n6)	3.54	1.8255
N-Arachidonoylglycine	1.62	0.6975
Malic acid	0.54	-0.8905
**Unstimulated-Positive mode**		
Palmitelaidic acid	7.57	0.0278
Hypogeic acid	7.57	0.0278
Palmitoleic acid	7.57	0.0278
Iodine	6.87	0.0174
DG(14:1(9Z)/16:1(9Z)/0:0)	4.20	0.0026
DG(16:1(9Z)/14:1(9Z)/0:0)	4.20	0.0026
Anabasine	2.35	0.0027
Butanone	2.25	0.0134
2-Furoic acid	1.88	0.0120
Choline	1.64	0.0074
2-Propyl-2,4-pentadienoic acid	0.63	0.0412
Pterin	0.60	0.0106
(9S,10R,12R)-2,3,9,10,11,12-Hexahydro-10-methoxy-2,9-dimethyl-1-oxo-9,12-epoxy-1H-diindolo[1,2,3-fg:3’,2’,1'-kl]pyrrolo[3,4-i][1,6]benzodiazocine-10-carboxylic acid, methyl ester	0.46	0.0054
**Unstimulated-Negative mode**		
PC(O-34:1)	3.87	0.0012
PC(O-18:1(9Z)/16:0)	3.87	0.0012
PC(O-18:1(11Z)/16:0)	3.87	0.0012
5-(3’,4’,5'-Trihydroxyphenyl)-gamma-valerolactone	1.38	0.0347
Orotic acid	0.50	0.0016

DG: Diacylglycerols; PC: Phosphatidylcholine.

### Metabolic pathways altered by the significant dysregulated metabolites

**Figure 2.**
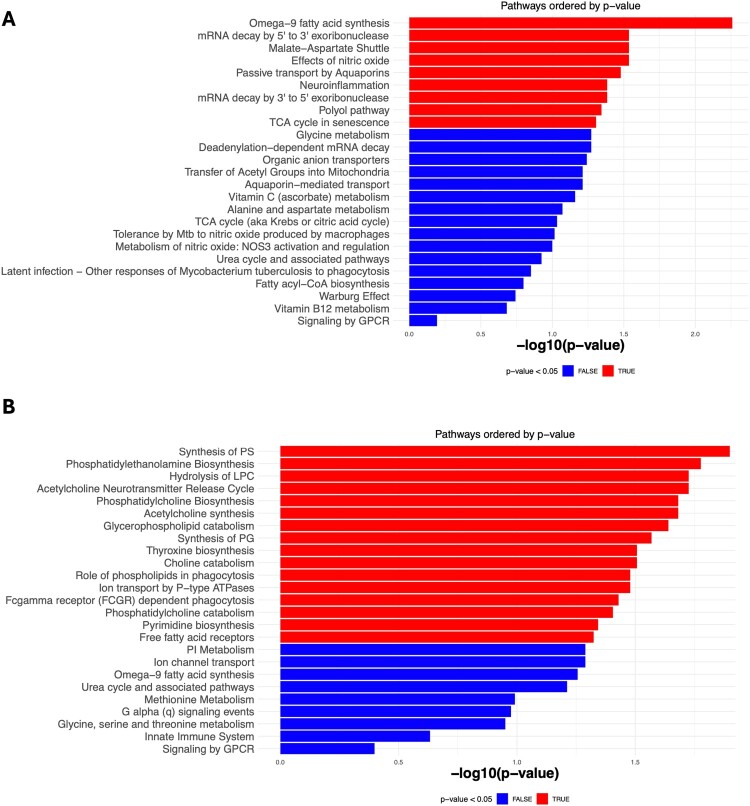
Overrepresentation Analysis of the Significantly Dysregulated Metabolites. Overrepresentation analysis of the significantly altered metabolites in (A) Stimulated supernatants and (B) Unstimulated supernatants. Red horizontal bars represent pathways which are significantly impacted (*p* value <0.05).

To interpret the functional significance of the dysregulated metabolites, we performed Over Representation Analysis (ORA). Since fatty acids and lipids were mainly dysregulated in these individuals, we observed a significant dysregulation majorly in the fatty acid biosynthesis and choline biosynthesis pathways. Apart from these, we also observed a significant alteration in the thyroxine biosynthesis, pyrimidine biosynthesis and mRNA degradation pathways (Figure 2).

### Potential biomarkers and biosignatures

**Figure 3.**
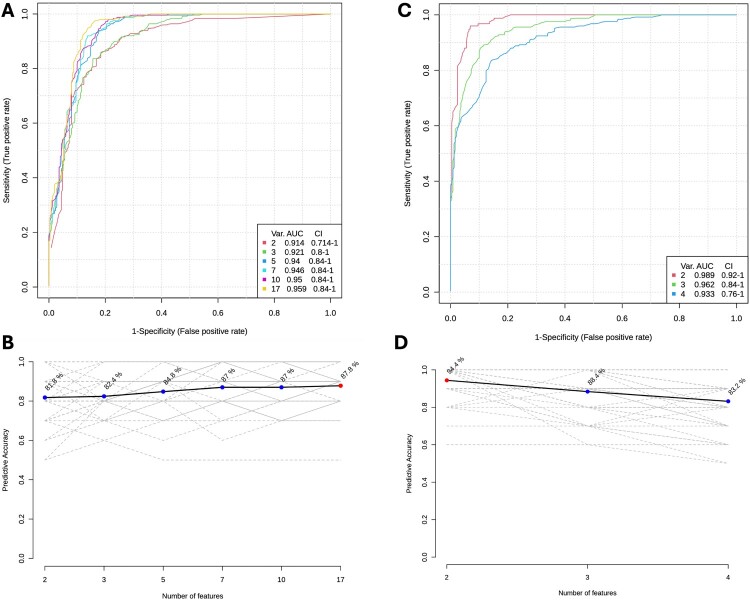
Biomarker prediction by Multivariate ROC Analysis of significantly altered metabolites in the stimulated supernatants. (A) Overview of all ROC curves created by MetaboAnalyst 6.0 from 6 different biomarker models derived from stimulated QuantiFERON supernatants in the positive mode considering different number of features (2, 3, 5, 7, 10, and 17) with their corresponding AUC values and confidence intervals. (B) Graph presenting the predictive accuracies of the 6 different biomarker models. The red dot specifies the highest accuracy for the 17-feature panel of model 6. (C) Overview of all ROC curves created by MetaboAnalyst 6.0 from 3 different biomarker models derived from stimulated QuantiFERON supernatants in the negative mode considering different number of features (2, 3 and 4) with their corresponding AUC values and confidence intervals. (D) Graph presenting the predictive accuracies of the 3 different biomarker models. The red dot specifies the highest accuracy for the 2-feature panel of model 1.

**Figure 4.**
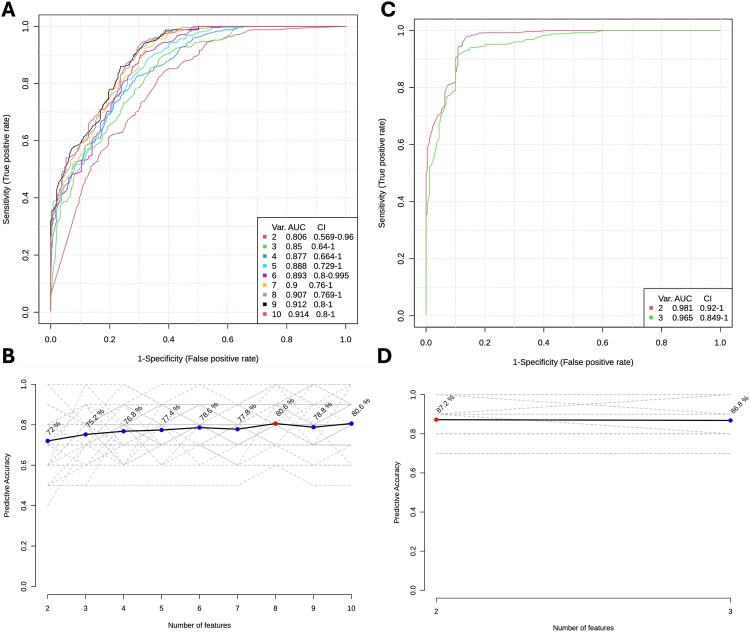
Biomarker prediction by Multivariate ROC Analysis of significantly dysregulated metabolites in the unstimulated supernatants. (A) Overview of all ROC curves created by MetaboAnalyst 6.0 from 6 different biomarker models derived from unstimulated QuantiFERON supernatants in the positive mode considering different number of features (2, 3, 4, 5, 6, 7, 8, 9 and 10) with their corresponding AUC values and confidence intervals. (B) Graph presenting the predictive accuracies of the 9 different biomarker models. The red dot specifies the highest accuracy for the 8-feature panel of model 7. (C) Overview of all ROC curves created by MetaboAnalyst 6.0 from 3 different biomarker models derived from unstimulated QuantiFERON supernatants in the negative mode considering different number of features (2 and 3) with their corresponding AUC values and confidence intervals. (D) Graph presenting the predictive accuracies of the 2 different biomarker models. The red dot specifies the highest accuracy for the 2-feature panel of model 1.

To determine the clinical utility of the significantly altered metabolites, we performed uni- and multi-variate analysis to identify the best possible metabolite biomarkers and biosignatures that are capable of predicting progression to active TB disease. The individual AUCs of the differentially regulated metabolites ranged between 0.56 and 0.96 in the stimulated supernatants and between 0.53 and 0.94 in the unstimulated supernatants (Table 3). In the stimulated supernatants, under negative mode, the combination of Malic acid and N-Arachidonoylglycine gave the maximum AUC of 0.98 with a predictive accuracy of 94.4%, while in the positive mode, a combination of all 17 metabolites gave the maximum predictive accuracy of 87.8% with an AUC of 0.96 (Figure 3). In the unstimulated supernatants, the combination of Orotic acid and phosphatidylcholines PC (O-34:1); PC (O-18:1(9Z)/16:0); PC (O-18:1(11Z)/16:0) gave the highest AUC of 0.98 with a predictive accuracy of 87.2% in the negative mode, whereas in the positive mode, a combination of 8 metabolites (2-Propyl-2,4-pentadienoic acid, (9S,10R,12R)-2,3,9,10,11,12-Hexahydro-10-methoxy-2,9-dimethyl-1-oxo-9,12-epoxy-1H-diindolo [1,2,3-fg:3’,2’,1'-kl] pyrrolo [3,4-i][1,6] benzodiazocine-10-carboxylic acid, Methyl ester, Pterin, Choline, Iodine and Fatty acid metabolites (Palmitelaidic acid, Palmitoleic acid, Hypogeic acid, Butanone and Anabasine) gave an AUC of 0.91 with a predictive accuracy of 80.6% (Figure 4).

**Table 3.** Diagnostic accuracy of the significantly dysregulated metabolites in the stimulated and unstimulated QuantiFERON supernatants of Progressors compared to Non-progressors

Metabolite	AUC	*p*-value
**Stimulated Supernatants – Positive mode**		
N-Methylnicotinium	0.98	0.0004
4,8-Methano-8aH-bisbenzofuro[3,2-e:2’,3'-g]isoquinoline-1,8a-diol,7-(cyclopropylmethyl)-5,6,7,8,9,14b-hexahydro-, (4bS,8R,8aS,14bR)-	0.89	0.0008
Diethanolamine	0.79	0.0094
N-Acetylserotonin sulphate	0.77	0.0155
Dimethyldithiophosphate	0.76	0.0582
PS(20:3(8Z,11Z,14Z)/18:0); PS(18:0/20:3(8Z,11Z,14Z))	0.75	0.0382
Pelargonic acid	0.75	0.0101
Dihydroresveratrol 4'-sulfate; Dihydroresveratrol 3-sulfate	0.67	0.0626
N,N,N-Trimethyl-L-alanyl-L-proline betaine; Leucylproline; Isoleucylproline	0.66	0.3995
Oxalic acid	0.65	0.9057
Nitrate	0.58	0.8536
Pyroglutamine; Dihydrothymine	0.55	0.7051
Butanol	0.55	0.3196
7-Methylguanosine	0.54	0.7053
3, 5-Tetradecadiencarnitine	0.54	0.6030
6-Methylthiopurine 5'-monophosphate ribonucleotide	0.53	0.6237
3-Methoxy-4-hydroxyphenylglycol glucuronide	0.51	0.9937
**Stimulated supernatants – Negative mode**		
Malic acid	0.77	0.0021
N-Arachidonoylglycine	0.65	0.1232
DG(18:2n6/0:0/20:3n6)	0.65	0.1210
Behenic acid	0.56	0.6867
**Unstimulated supernatants – Positive mode**		
2-Propyl-2,4-pentadienoic acid	0.87	0.0001
(9S,10R,12R)-2,3,9,10,11,12-Hexahydro-10-methoxy-2,9-dimethyl-1-oxo-9,12-epoxy-1H-diindolo[1,2,3-fg:3’,2’,1'-kl]pyrrolo[3,4-i][1,6]benzodiazocine-10-carboxylic acid, methyl ester	0.85	0.0032
Pterin	0.82	0.0006
Choline	0.80	0.0008
Iodine	0.73	0.0239
Palmitelaidic acid; Palmitoleic acid; Hypogeic acid	0.72	0.0868
Butanone	0.66	0.5822
Anabasine	0.64	0.1389
2-Furoic acid	0.59	0.6092
DG (14:1(9Z)/16:1(9Z)/0:0); DG(16:1(9Z)/14:1(9Z)/0:0)	0.57	0.1202
**Unstimulated supernatants – Negative mode**		
Orotic acid	0.96	0.0001
PC(O-34:1); PC(O-18:1(9Z)/16:0); PC(O-18:1(11Z)/16:0)	0.90	0.0130
5-(3’,4’,5'-Trihydroxyphenyl)-gamma-valerolactone	0.53	0.3846

AUC: Area under the Curve.

## Discussion

Infection by *Mtb* causes a major reprogramming of the host metabolic pathways to bolster the immune response leading to dysregulated production of various metabolites. In this preliminary study, we identified 21 metabolites that were significantly different in the stimulated supernatants and 18 metabolites that were significantly different in the unstimulated supernatants of Progressors and Non-progressors.

N-Acetylserotonin sulfate, a key metabolite of the tryptophan metabolism pathway, was found to be present at significantly elevated levels in the Progressors. Tryptophan has been previously reported as a biomarker for tuberculosis disease progression [12]. Many metabolites of fatty acid metabolism including Behenic acid, Palmitelaidic acid, Hypogeic acid, Palmitoleic acid, and lipids like Diglycerides DG (14:1(9Z)/16:1(9Z)/0:0), DG (16:1(9Z)/14:1(9Z)/0:0), Choline, Phosphatidylcholines like PC (O-34:1), PC (O-18:1(9Z)/16:0) and PC (O-18:1(11Z)/16:0) were found to be present at highly abundant levels in Progressors compared to Non-progressors. *Mtb* is known to utilize host fatty acids and lipids for its survival and for building its lipid-rich cell wall [20]. Studies have also shown that *Mtb* rewires the fatty acid and lipid metabolism of the host macrophages to create a hospitable niche for its intracellular survival [21]. Another important metabolite that was seen in high abundance in Progressors was 3, 5-Tetradecadiencarnitine, an acylcarnitine which is a fatty acid intermediate. Weiner *et al.* had also observed increased levels of plasma metabolites of the fatty acid metabolism pathway including acylcarnitines and 3-hydroxybutyrate in their cohort of TST/QFT converters [11].

Increased abundance of nitrate seen in Progressors favours the survival of *Mtb* inside the oxygen-deficient granuloma, as *Mtb* can initiate the nitrate reductase activity [22,23]. This was evident from the pathway analysis which identified three important pathways related to nitric oxide metabolism that were being upregulated by these metabolites.

N-Methylnicotinium, a nicotine derivative was identified among the significantly altered metabolites in this cohort. But interestingly, the level of N-Methylnicotinium was significantly downregulated in the Progressors. This observation correlates with Weiner *et al*’s observation of lower levels of another nicotine derivative, cotinine, in Progressors at time points closer to TB diagnosis [10]. The significance of this observation is yet to be explored. We also observed a 2.35-fold increase of Anabasine, a nictotine analog in Progressors. Anabasine is a minor alkaloid found in tobacco and has been used as a biomarker of tobacco smoking exposure [24,25]. It is also widely used as pesticides [26].

Taken together, it may be understood that metabolites of the fatty acid and lipids are mainly dysregulated in Progressors. We went on to evaluate the diagnostic performance of these metabolites for their utility as clinical biomarkers/biosignatures. We found that the combination of Malic acid and N-Arachidonoylglycine in the stimulated supernatant gave the maximum AUC of 0.98, while the combination of Orotic acid and the phosphatidylcholines PC (O-34:1), PC (O-18:1(9Z)/16:0), PC (O-18:1(11Z)/16:0) in the unstimulated supernatants gave the maximum AUC of 0.99. Weiner *et al.* investigated the serum and plasma metabolome of Progressors and Non-progressors in Africa and derived a model with six metabolites (Cortisol, Glutamine, Cotinine, Kynurenine, Histidine and Mannose) that gave a diagnostic accuracy ranging from 0.73 to 0.92 in the proximate samples (<5 months to TB diagnosis) of the validation data set [10]. A 25-metabolite signature (MetabAD) that differentiated Progressors from Healthy controls with an AUC of 0.7 in samples within 6 months of TB diagnosis was also reported. This signature when combined with the ACS-CoR transcriptional signature showed an improved AUC of 0.8 [12]. More recently, Pantothenate (Vitamin B5) along with other metabolites of inflammation (9-HETE), fatty acid metabolism (acylcarnitines and 3-hydroxybutyrate) and bile acids (glycochenodeoxycholate sulfate and taurocholenate) were reported as potential biomarkers in TST/QFT converters vs non-converters [11]. Here, we also identified a set of highly promising TB-specific metabolic signatures with high predictive accuracy which warrants further validation using targeted metabolomics in diverse cohorts so that they can be recommended as useful biomarkers for predicting risk of progression to TB.

There are some limitations in our study. Firstly, the size of our cohort was small, but its greatest strength was the inclusion of well-characterized, systematically followed-up HHCs at 6 monthly intervals, as well as clearly delineated controls. Secondly, the identified metabolites are putative since the identifications are at the precursor level and hence requires further validation in multiple independent cohorts to validate their performance. It should be also noted that, since we have used samples at time points closest to their TB breakdown (3-6 months), these metabolite signatures have the potential to serve as short-term risk predictors. Longitudinal profiling of the Progressors at other farther time points is necessary to understand the performance of these markers at time points well ahead of disease progression.
